# Enhancing the value of meat inspection records for broiler health and welfare surveillance: longitudinal detection of relational patterns

**DOI:** 10.1186/s12917-021-02970-2

**Published:** 2021-08-18

**Authors:** S. N. Buzdugan, P. Alarcon, B. Huntington, J. Rushton, D. P. Blake, J. Guitian

**Affiliations:** 1 Veterinary Epidemiology, Economics and Public Health Group, Pathobiology and Population Sciences, Royal Veterinary College, Hawkshead Lane, Hertfordshire AL9 7TA North Mymms, UK; 2 Liverpool Science Park, Innovation Centre 2, 146 Brownlow Hill, L3 5RF Liverpool, UK; 3 Epidemiology and Population Health, Liverpool University, Brownlow Hill, L69 7ZX Liverpool, UK; 4 Pathobiology and Population Sciences, Royal Veterinary College, North Mymms, UK

**Keywords:** Meat inspection data, Machine learning, Relational patterns, Monitoring, Surveillance, Broiler chickens

## Abstract

**Background:**

Abattoir data are under-used for surveillance. Nationwide surveillance could benefit from using data on meat inspection findings, but several limitations need to be overcome. At the producer level, interpretation of meat inspection findings is a notable opportunity for surveillance with relevance to animal health and welfare. In this study, we propose that discovery and monitoring of relational patterns between condemnation conditions co-present in broiler batches at meat inspection can provide valuable information for surveillance of farmed animal health and welfare.

**Results:**

Great Britain (GB)-based integrator meat inspection records for 14,045 broiler batches slaughtered in nine, four monthly intervals were assessed for the presence of surveillance indicators relevant to broiler health and welfare. *K*-means and correlation-based hierarchical clustering, and association rules analyses were performed to identify relational patterns in the data. Incidence of condemnation showed seasonal and temporal variation, which was detected by association rules analysis. Syndrome-related and non-specific relational patterns were detected in some months of meat inspection records. A potentially syndromic cluster was identified in May 2016 consisting of infection-related conditions: pericarditis, perihepatitis, peritonitis, and abnormal colour. Non-specific trends were identified in some months as an unusual combination of condemnation reasons in broiler batches.

**Conclusions:**

We conclude that the detection of relational patterns in meat inspection records could provide producer-level surveillance indicators with relevance to broiler chicken health and welfare.

## Background

The value of meat inspection data for surveillance has been demonstrated in several countries [1]. In Great Britain (GB), despite the statutory requirement for collection of nationwide meat inspection data, they are not routinely and comprehensively used for animal health and welfare surveillance. Abattoir data, which are characterised by high throughput and wide coverage, could be added to current surveillance systems to inform improvements in animal health and welfare [2]. Despite these characteristics, the potential for the use of abattoir data for surveillance could be undermined by some limitations. Firstly, there is a need for improved communication of animal health and welfare findings between abattoirs and producers, which might currently be limited at the individual producer level [3]. Secondly, although the conditions diagnosed at meat inspection are relevant to animal health and welfare, they are pre-diagnostic and require interpretation for recognition of specific diseases [2, 3].

The importance of feedback on meat inspection findings has been recognised in the development of health schemes available for some production animals [4, 5]. To our knowledge, no such schemes are available in broiler production. While feedback on abattoir data could be facilitated by the flow of information beneficial to integrated broiler production, the feedback might be restricted to high prevalence conditions [6]. Data analysis and interpretation are also required to ascertain the importance of abattoir findings for specific population health concerns [3, 6, 7].

The use of health-related pre-diagnostic data has been shown to provide early recognition of general disease clusters in human populations, supporting rapid response [8, 9]. One approach to early recognition of disease at the population level is syndromic surveillance [10]. Syndromic surveillance (SyS) is real-time (or near real-time) collection, analysis, interpretation, and dissemination of health-related data for the identification of specific signs, or groups of signs, in space and time to enable the early identification of potential human or animal health threats that require public and/or animal health action [11, 12]. More specifically, syndromic surveillance can be used to establish and then monitor health-related indicators rather than specific disease events [13, 14]. Surveillance indicators can be specific or non-specific, relating to distinct syndromes or diseases and identifying trends of disease outbreaks [10, 13]. Initially developed for early detection of large scale outbreaks of recognised diseases, applications for syndromic surveillance have expanded to include emerging diseases [13] and awareness of animal health throughout the production chain [15].

Previous studies evaluating the suitability of abattoir condemnation data for syndromic surveillance have focused on the identification of the most appropriate syndromic indicators, using expert knowledge or trends in factors associated with specific diagnosis [16–18]. An alternative to a predefined case or an outcome is a data-driven approach focused on identifying and establishing new and unexpected syndromic indicators for surveillance purposes [19]. Unsupervised machine learning algorithms that allow real-time identification of patterns in the data without the need to pre-define a number of classes (in this case groups of conditions) are, *a priori*, particularly suitable for this task.

To our knowledge, it remains unknown whether it is possible to detect unspecified surveillance indicators in broiler chicken abattoir data over relatively short time intervals. In this study, we use routinely collected producer-level broiler meat inspection data on batch-level co-morbidities to investigate the incidence of relational patterns between broiler condemnation categories, and we evaluate them as non-specific surveillance indicators.

## Methods

### Data management

Data were collected from a commercial broiler integrator located in England for 2015–2017. The data comprised of batch-level condemnation records for twenty-three broiler health and welfare-related conditions resulting from compulsory abattoir procedures; the list of conditions and their codes used for analysis are presented elsewhere [20]. Conditions related to processing insufficiencies (e.g. overscald) were not considered for analysis.

In the dataset, counts for individual condemnation categories were recorded per batch of slaughtered broilers, where a batch was a group of broilers from the same farm and barn delivered to the slaughterhouse on the same vehicle. Counts were transformed into percentages for cluster analyses and were categorised for association rules analysis. Data categorisation for each condemnation reason was carried out using median condemnation counts as the cut-off. Batches with a number of rejected carcasses above the median value were coded as 1; batches with a number of rejections at or below the median were coded 0.5; batches where no carcasses were condemned were coded as 0. Median values were calculated for each condemnation category using data on batches with at least one condemnation. Thus, the batch-level diagnosis of morbidities was categorised into null, low or high condemnation categories.

The data were divided and analysed at monthly intervals to detect and evaluate the incidence of relational patterns. Data for three separate months were selected in each study year due to the large volume of meat inspection records and the consequential computational limitations, and to represent seasonal trends in condemnation. Analyses were therefore conducted on nine individual one-month intervals: January, May, and September of 2015, 2016 and 2017.

### Data analysis

Unsupervised machine learning methods were used to discover underlying structure(s) within the condemnation data that would not be otherwise visible. These methods were cluster analysis and association rules mining. These unsupervised machine learning methods are not predictive, unlike supervised machine learning methods, but inferential, and thus, their goal is pattern detection assuming that the trends of the past will continue. Analyses were conducted independently on nine, one-month integrator broiler abattoir datasets to detect and evaluate relational structures available in meat inspection data longitudinally. Data analyses were carried out in R (v.3.5.1).

### K-means clustering

*K*-means cluster analysis was used to identify subsets of broiler batches with similar condemnation profiles and to group them into clusters. The clustering patterns were compared between the time intervals. A detailed description of the *k*-means clustering method is available elsewhere [21]. Briefly, the Euclidian distance-based *k*-means clustering algorithm used for analysis minimised the sum of distances from all data points to a cluster centroid. Therefore, the minimal distance was selected over all clusters until the distance could not be decreased any further. As the *k*-means algorithm required a predefined number of clusters, *k*, which corresponded to the number of centroids for the variable’s assignment, we determined *k* by successively changing the value by an increment of one. The smallest number of clusters that explained the highest amount of variance was selected [22]. *K*-means analysis was performed using the R package *factoextra* [23].

### Hierarchical clustering

The aim for hierarchical cluster analysis was to identify groups of condemnation categories that were similar across broiler batches. In contrast to *k*-means cluster analysis that clustered batches by condemnation profile, hierarchical clustering was used to cluster variables.

We clustered variables using a correlation-based agglomerative hierarchical clustering approach to identify clusters of conditions with the same overall profile, regardless of magnitude. The cluster was identified based on homogeneity, which was defined as the sum of the squared correlation between variables and the centre of the cluster measured by squared Pearson’s correlation coefficient [24].

The optimal number of clusters was selected for each monthly interval after a hierarchy of nested clusters, a hierarchical dendrogram, was built. Dendrogram structure was assessed visually and more formally using the stability of variable partitions with Rand indices as proposed by Hubert and Arabie (1985), and adapted by Chavent et al. (2011). The hierarchical clustering of variables was carried out using the *ClustOfVar* R package [24].

### Association rules analysis

The aim of the association rules analysis was to identify patterns in sets of condemnation conditions that frequently occurred together in a high proportion of broiler batches within each of nine months of abattoir records. Although the discovery and interpretation of individual rules are often central in conducting association rules analysis, the main focus here was to discover patterns.

Association rules analysis was carried out in two steps: a selection of constraints specified as support (i.e. frequency of a rule in a dataset) and confidence (i.e. conditional probability between conditions in a rule, antecedent and a consequent) values using all data for nine months; followed by an analysis of monthly condemnation records using the selected constraint values [25, 26]. The selection of thresholds for support and confidence of the rules was conducted by gradually increasing support and confidence values for subsequent models until a relatively small number of rules were generated (n < 55). This was achieved at a support level of 0.25 (25 %) and a confidence level of 0.5 (50 %), to exclude generation of a high number of irrelevant rules [27]. Thus, analysis focused on the detection of associations of the most prevalent conditions, in contrast to very infrequently observed groups of condemnations.

Association rules generated by the nine models were assessed after removing redundant rules using the Bayardo improvement method, where a rule was removed when a more general rule with the same consequent and the same or higher confidence was available [26]. The final sets of rules for each of the nine, one-month intervals were evaluated for stability by comparing the presence of specific rules between subsequent months. A 10 % change in the confidence in an association rule present in two subsequent time intervals was selected as an outcome. An increase in confidence between prevalent condemnation conditions, indicating an increase in their predictive ability, was selected as an outcome that could be relevant to the formation of a syndromic cluster [28]. Therefore, the monthly pattern of stable rules was identified as a target outcome for analysis. The analysis was carried out using the R package *Arules* [29].

## Results

### Data summary

Condemnation data from 55,918 broiler batches slaughtered by the integrator were collected between 2015 and 2017. Data from January, May and September were extracted for analysis from each year, producing a dataset with data from nine months which included 14,045 broiler batches (~ 25 % to the total). Median broiler batch size was circa 5500, with a minimum of 700 and a maximum of 11,000. In total, 77,640,763 broilers were slaughtered during the nine months used for analysis (Table 1). As can be inferred from Table 1, batch-level data had a high level of overlap between condemnation categories.

**Table 1:** Prevalence of individual condemnation conditions at the batch or individual broiler carcass level for twenty-three condemnation categories collected in January, May and September 2015–2017. In total 14,045 batches were considered here, including 77,640,763 individual broilers. Note*: ‘other farm’ category included Oregon and muscle myopathies

Condemnation condition	Code	Condemnation outcome type	Number of batches with at least one carcass condemned (%)	Number of carcasses with individual condemnations (%)
Abnormal colour	ABN	whole carcass	13,843 (98.56 %)	328,611 (0.42 %)
Ascites	AST	whole carcass	13,906 (99.01 %)	379,384 (0.49 %)
Bruising	BRU	whole carcass	8457 (60.21 %)	19,645 (0.03 %)
Cellulitis	CEL	whole carcass	11,469 (81.65 %)	91,059 (0.12 %)
Dead On Arrival	DOA	whole carcass	13,123 (93 %)	101,266 (0.13 %)
Dermatitis	DER	whole carcass	6082 (43.3 %)	26,890 (0.03 %)
Emaciation	EMA	whole carcass	3088 (22.19 %)	6684 (0.01 %)
Evisceration Runts	EVR	whole carcass	4598 (32 %)	33,540 (0.04 %)
Foliculitis	FOL	whole carcass	1 (0.01 %)	6 (0 %)
Heard Breast	HB	whole carcass	11,031 (78 %)	121,045 (0.16 %)
Hepatitis	HEP	whole carcass	96 (0.68 %)	405 (0 %)
Intake runts	IR	whole carcass	2351 (16 %)	9711 (0.01 %)
Jaundice	JND	whole carcass	1049 (7.46 %)	1442 (0 %)
Joint lesions	JNT	whole carcass	67 (0.48 %)	155 (0 %)
Partial - Hearts	Prtheart	partial	7920 (56.39 %)	34,440 (0.04 %)
Partial - Livers	Prtliv	partial	12,318 (87.7 %)	102,530 (0.13 %)
Pericarditis	PCS	whole carcass	2538 (18.07 %)	5319 (0.01 %)
Perihepatitis	PHS	whole carcass	11,578 (82.44 %)	71,144 (0.09 %)
Peritonitis	PTS	whole carcass	2689 (19.15 %)	4328 (0.01 %)
Other farm*	FOT	whole carcass	1450 (10.32 %)	16,901 (0.02 %)
Respiratory conditions	RES	whole carcass	3 (0.02 %)	4 (0 %)
Skin conditions	SKN	whole carcass	1115 (7.94 %)	5055 (0.01 %)
Tumours	TUM	whole carcass	10,993 (78.27 %)	36,050 (0.05 %)

**Fig. 1:**
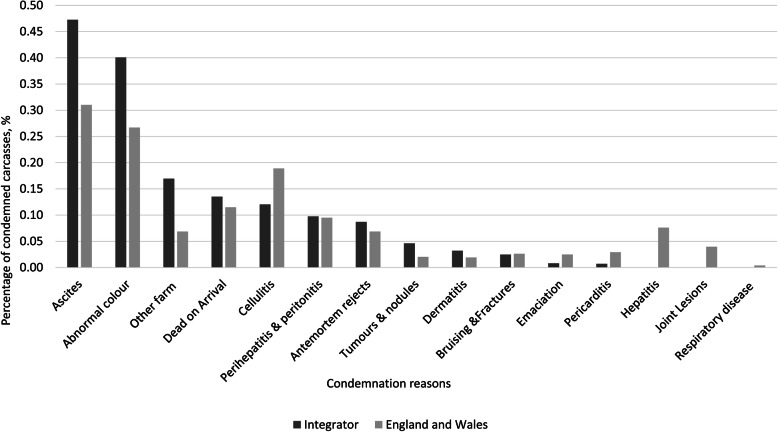
Comparison of the occurrence of condemnation conditions in individual broiler chickens for 2015, 2016 and 2017. Data from the integrator are shown, in dark grey. Data from the FSA (England and Wales) are presented in light grey

The integrator processed on average 8.5 million broilers every month during the sampling period, representing ~ 13 % of broilers processed during the same period in England and Wales [30]. Figures from the Food Standards Agency (FSA), a department of the Government of the United Kingdom responsible for protecting public health in relation to food in England, Wales and Northern Ireland, were accessed for comparison. In both integrator and national records, ascites was the leading cause of condemnation, followed by abnormal colour (Fig. 1). Nationally, cellulitis was the third most common cause of condemnation in the FSA data [31], although this ranked lower in the integrator records, where ‘Other farm’ and ‘Dead on arrival’ were more common.

**Fig. 2:**
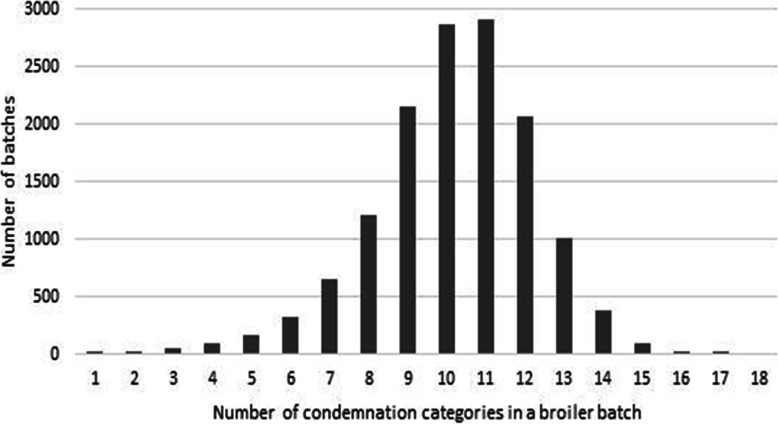
Number of co-diagnosed condemnation category conditions per broiler batch, sampling 14,045 batch-level records over nine months during 2015–2017

Consideration of batch-level integrator data revealed that 13,994 of 14,045 batches (99 %) presented with two or more condemnation reasons. Multiple reasons were common, with a minimum of one and maximum of 17 conditions per batch (Fig. 2). Just 1 % of batches presented with a single reason for condemnation. The mean number of conditions reported in a batch was 10 (Interquartile range: 9–11) (Fig. 2).

### Monthly condemnation frequency

**Fig. 3:**
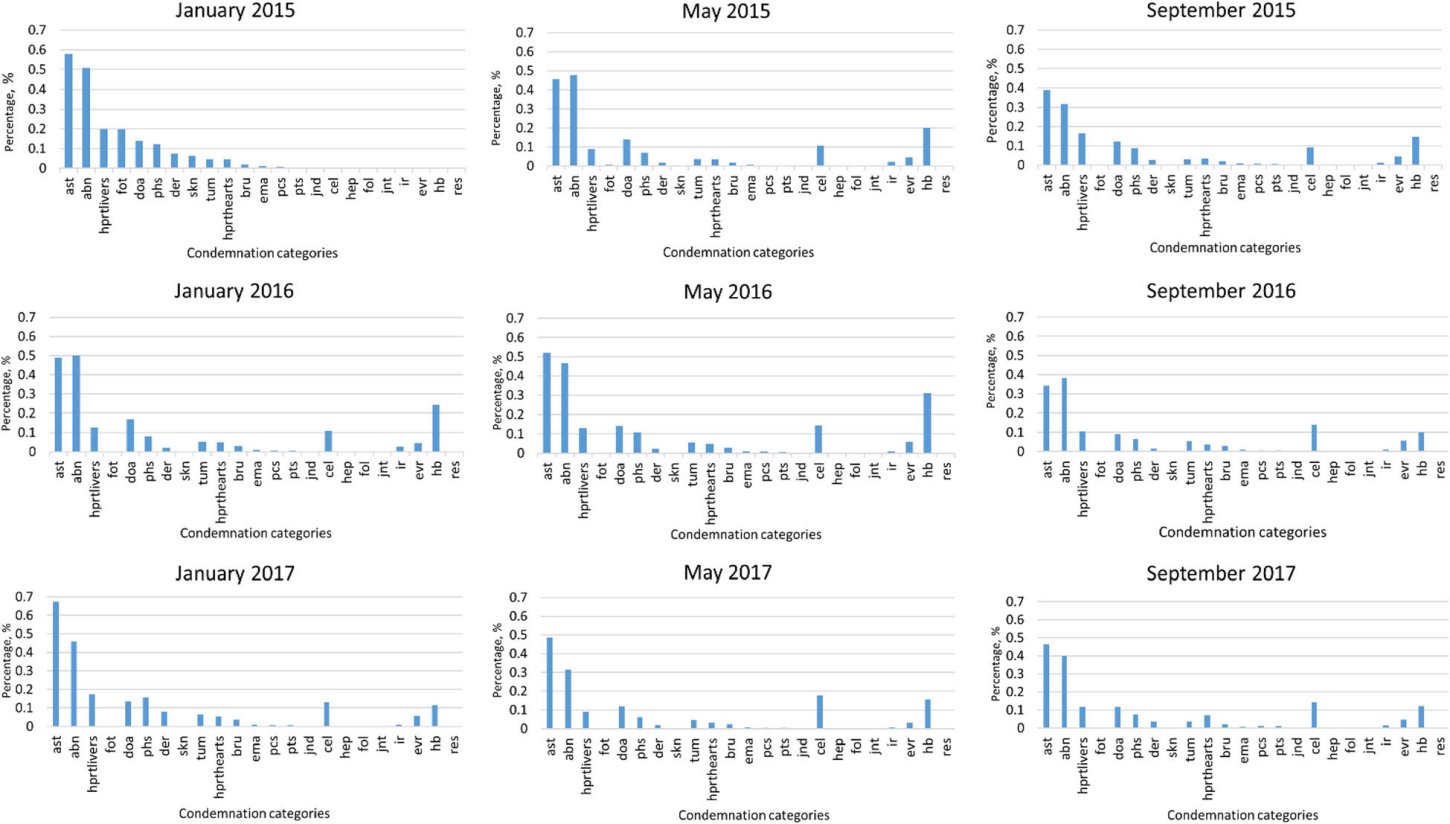
Monthly percentage of whole carcass condemnation at individual chicken-level for health and welfare-related conditions in the integrator data, sampling 14,045 batches. Partial rejects of liver and heart were also included in the analysis

The monthly incidence of individual condemnation categories calculated over the study period showed similar trends. Ascites and abnormal colour were the most common reasons for whole carcass condemnation (Fig. 3). Greater variation was detected among other condemnation conditions, with examples such as hard breast and cellulitis common.

**Fig. 4:**
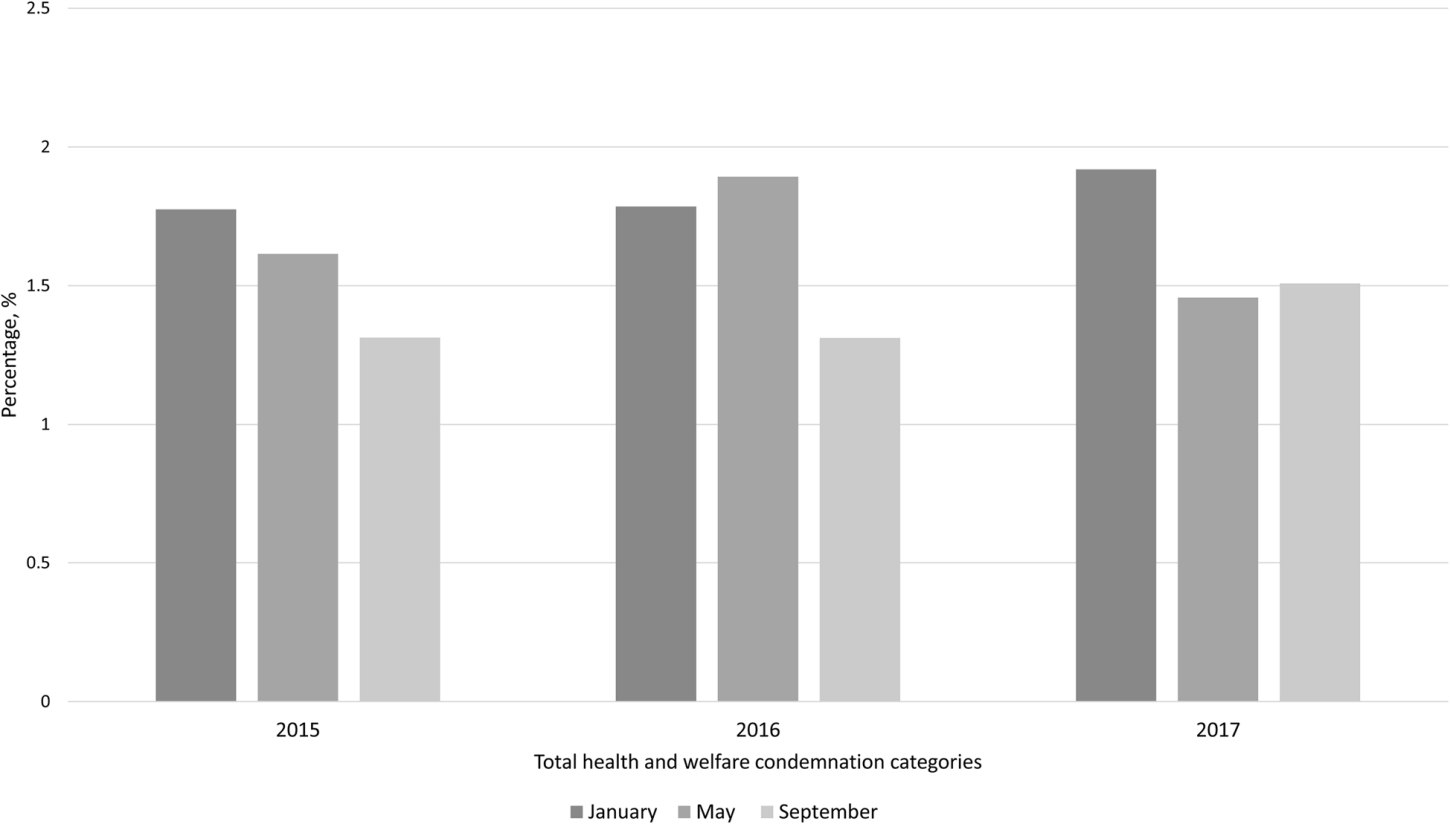
Monthly percentage of condemnation for health and welfare-related conditions for January, May and September (presented from dark grey to light) 2015–2017 in the integrator data, sampling 14,045 batches

Monthly all-cause condemnation incidence demonstrated a distinct seasonal variation during the three years of the study (Fig. 4). From the three months studied each year, January consistently presented the highest incidence of all-cause condemnation. September was generally lowest except for 2017 when the figures for May were below that month’s average.

### Results of the K-means cluster analysis

**Fig. 5:**
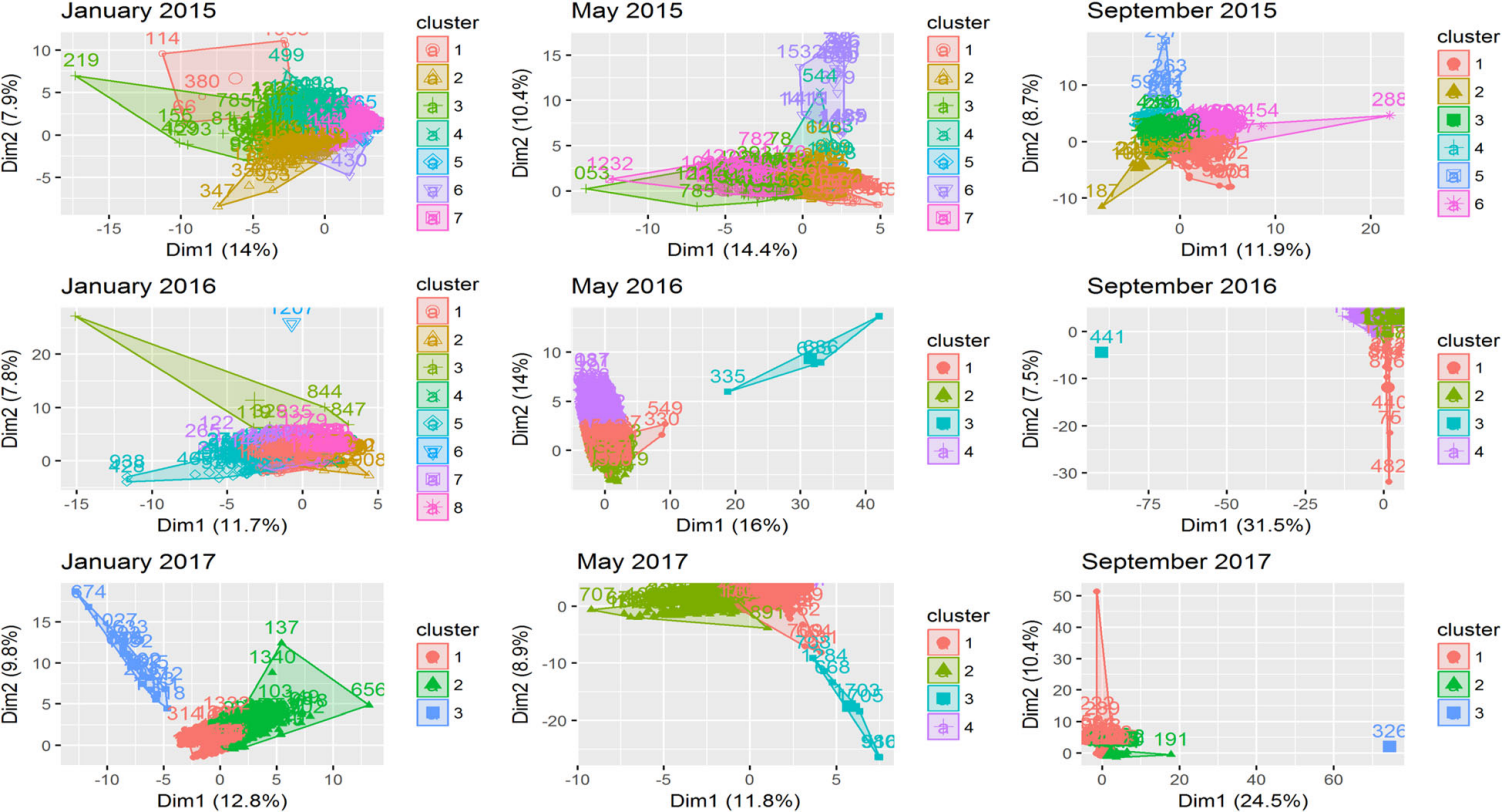
Clustering of broiler batches based on similarity in incidence of condemnation reasons using k-means clustering. The colour of each observation (broiler batch) indicates a cluster to which it was assigned. Distinct clusters are identified in different colours, while batch I.D. numbers indicate specific cluster membership. Clusters represented by the same colour in each monthly interval do not correspond to the same condemnation profile

Condemnation profiles for broiler batches processed in January, May or September 2015–2017 were not constant (Fig. 5). Clusters overlapped in all monthly condemnation records. On average, less than 40 % of the variation between batches was explained by clustering in each monthly interval. Exceptions included May 2016 and January 2017, both of which presented distinct clusters that were unique from the other clusters (Fig. 5). In May 2016 batches with a high number of carcasses condemned for dermatitis, hepatitis, Oregon, myopathies, and joint lesions were grouped into an outlier cluster.

The highest number of clusters were identified in the first four one-month intervals (i.e. January, May and September 2015, January 2016), indicating higher heterogeneity between broiler batches. Thereafter, four or fewer clusters described batch-level condemnation reasons between batches, indicating more homogeneous condemnation conditions across integrator farms (Fig. 5).

### Results of hierarchical cluster analysis

**Fig. 6:**
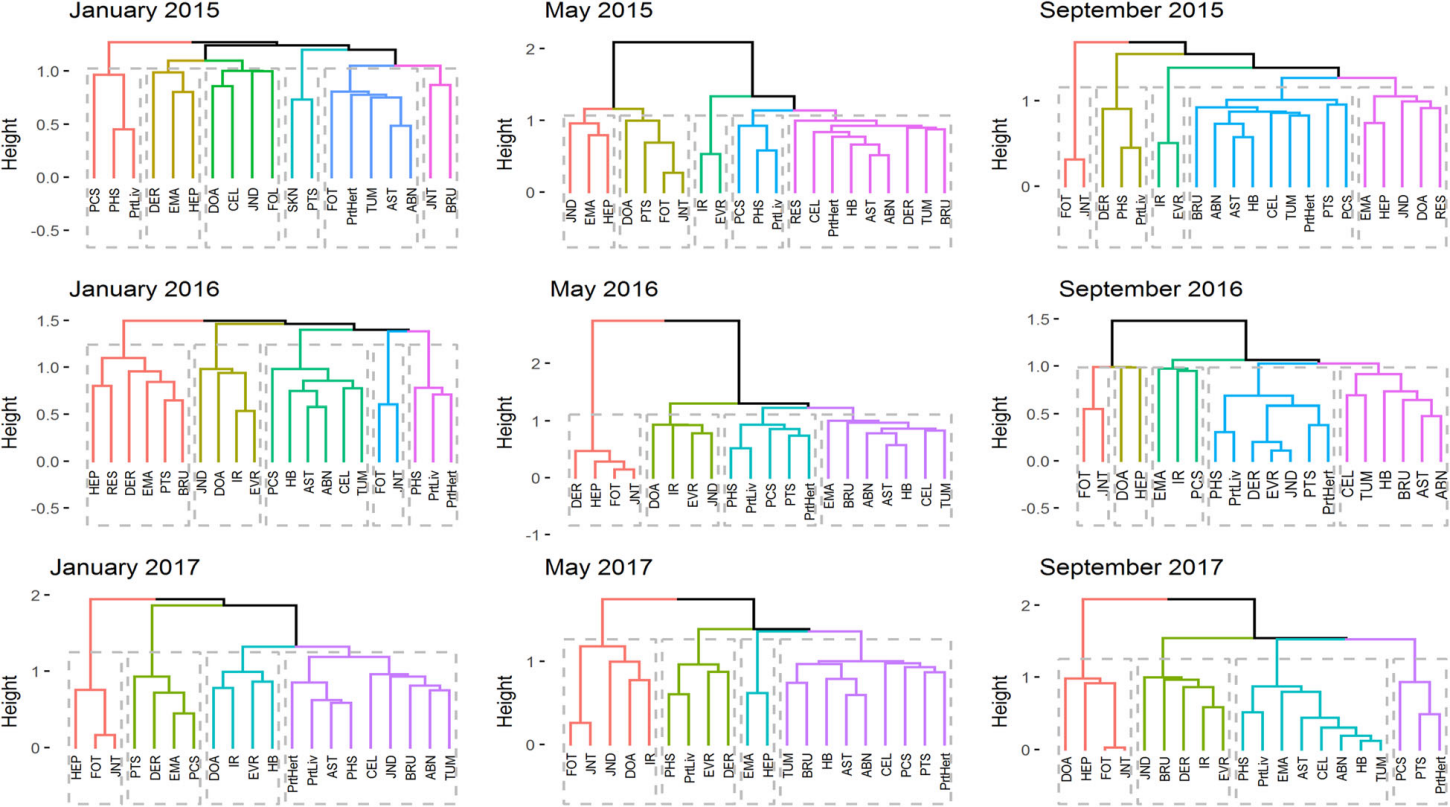
Correlation-based hierarchical clustering trees of condemnation reasons for nine abattoir datasets. Conditions with strong correlations are situated nearer on the branches of the dendrogram.*For a list of abbreviations for condemnation categories please see Table 1, for a detailed description please see [20]

In common with results from *k*-means clustering, patterns of condemnation reasons detected by hierarchical clustering also varied between months (Fig. 6). Nonetheless, a small number of condemnation categories were consistently paired including (i) partial rejection of liver (Prtliv) and whole carcass condemnations for perihepatitis (PHS), and (ii) ascites (AST) and abnormal colour (ABN) or hard breast (HB) (Fig. 6). In May 2016, conditions that were likely to relate to the presence of infectious pathogens such as pericarditis (PCS), perihepatitis (PHS), peritonitis (PTS), and partial liver (Prtliv) and heart rejects (Prtheart), showed correlation and were found to cluster (Fig. 6).

An agreement between *k*-means and correlation-based hierarchical clustering methods was identified. The outlier cluster composed of condemnation categories for dermatitis (DER), hepatitis (HEP), Oregon, myopathies (FOT) and joint lesions (JNT) identified in May 2016 by *k*-means analysis was confirmed by correlation-based hierarchical clustering (Fig. 6, May 2016 in red).

### Results of association rules analysis

**Fig. 7:**
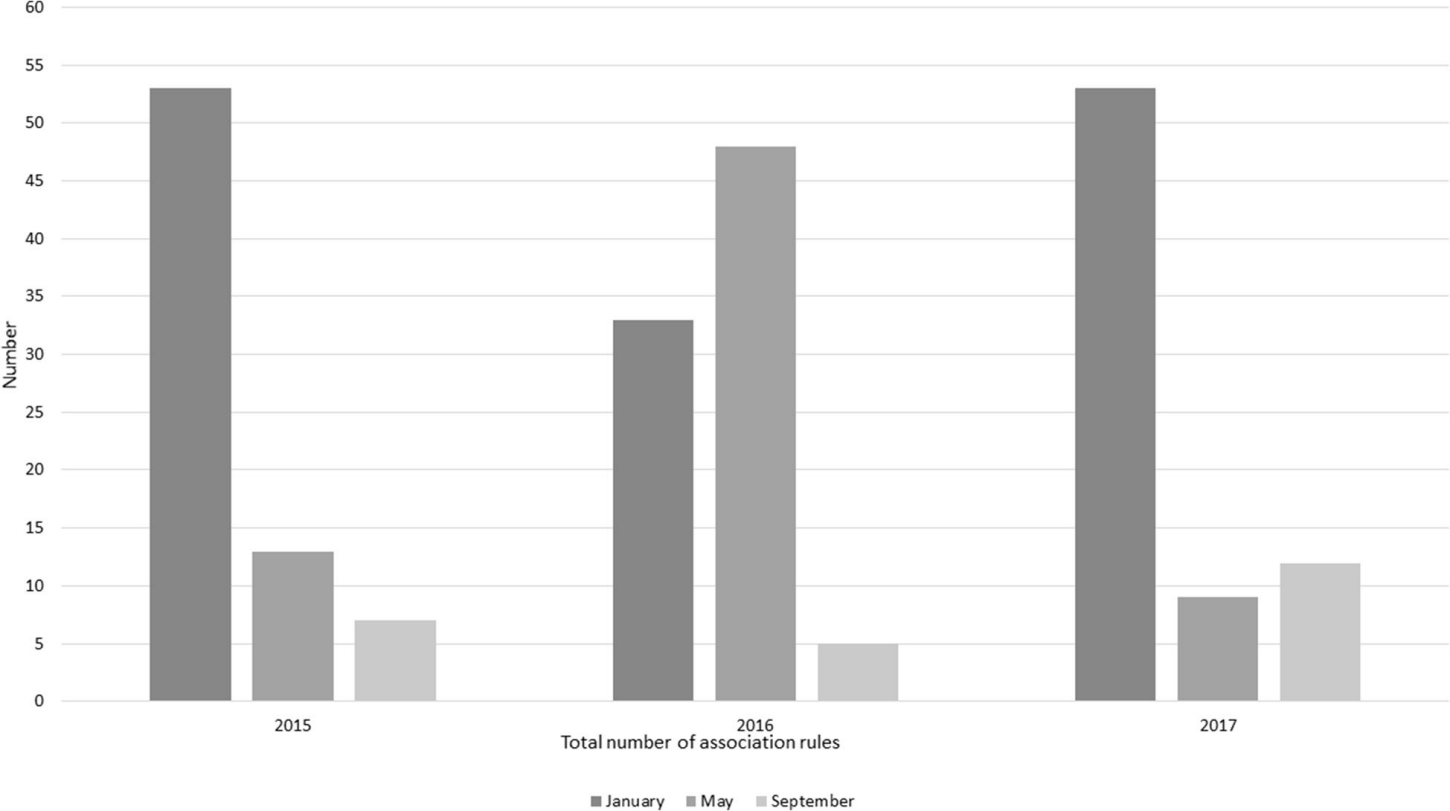
The total number of association rules generated for January, May and September (presented from dark grey to light) 2015–2017, using 25 % support and 50 % confidence on nine datasets of the integrator abattoir condemnation records

Two patterns were detected by association rules analysis: a trend in the number of association rules in months studied, and a number of stable rules that increased their confidence over time. A higher number of prevalent rules passed the specified thresholds for support and confidence in January compared to September of every year (Fig. 7). This pattern corresponded to the incidence of all-cause condemnations presented in Fig. 4 in every study month except May 2016, when a higher number of association rules passed the thresholds for support and confidence (*n* = 47) (Fig. 7).

**Fig. 8:**
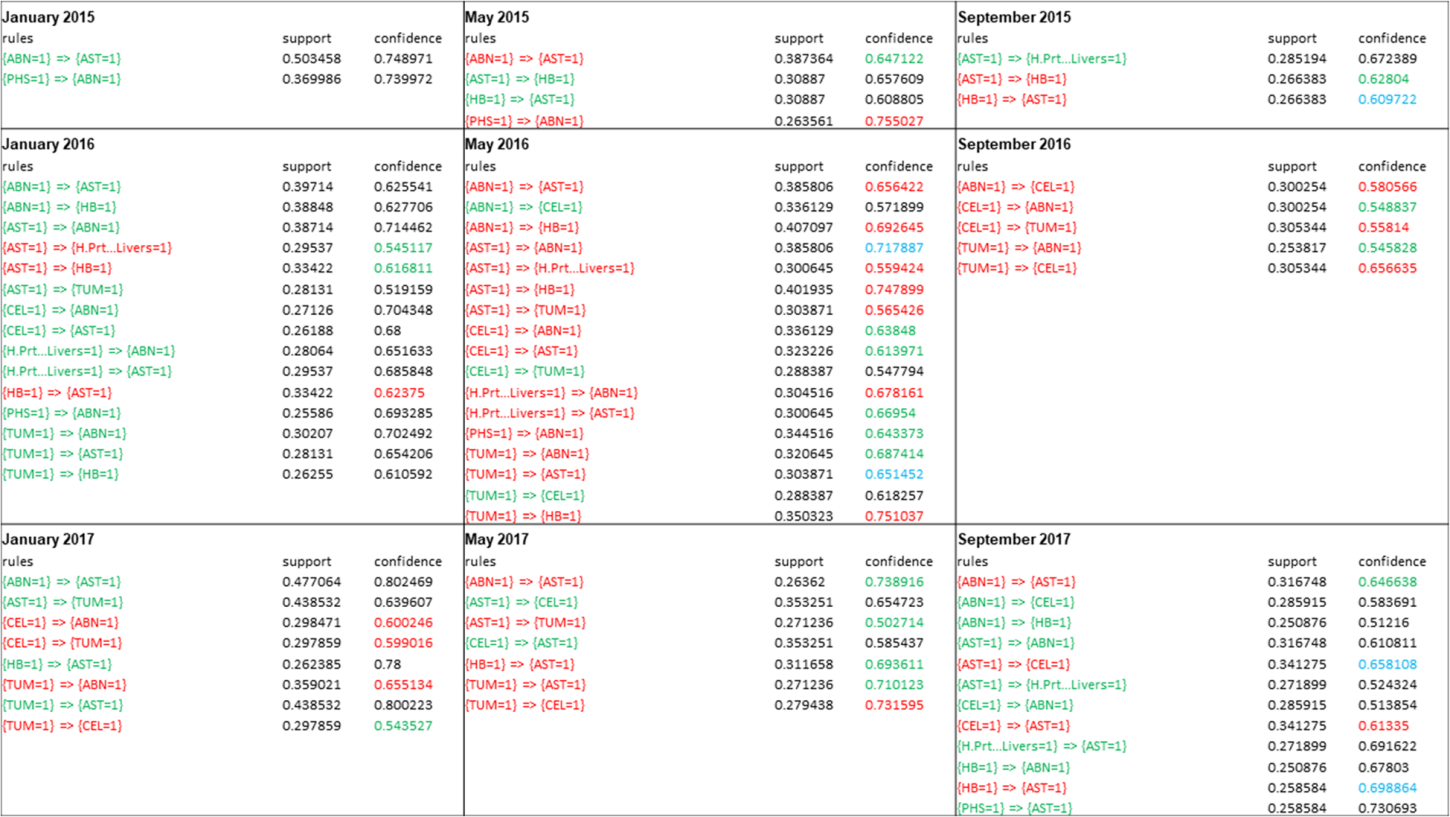
Association rules that were stable from the preceding sampled month, generated using 25 % support and 50 % confidence on nine datasets of abattoir condemnation records. Rules in green and italics font were not observed in the previous time interval; rules in red and underlined were observed in the previous sampled month (i.e. are stable). For instance, a rule with abnormal colour (ABN) and ascites (AST) was detected in January 2015 with 50 % support and 74 % probability of the two conditions being observed together; this association had lower probability (64 % indicated by green colour) in May 2015. A change in confidence value from the preceding month is indicated by different background colours and superscript letters: confidence that decreased between the two subsequent time intervals is indicated in green, an increase in confidence is indicated in red, while confidence values marked in blue did not change. *For a list of abbreviations for condemnation categories please see Table 1 [20]

Another pattern was detected in monthly meat inspection records when the stability of the rules was assessed by comparing the presence of individual rules in a given month to the previous one; their repeatability was variable. The highest number of stable rules was between January, May and September 2016. In May 2016, 14 rules were retained from January 2016, seven of which had increased their value of confidence (Fig. 8). Finally, discovered rules indicated associations between ascites and abnormal colour, as well as ascites and hard breast or tumours in the majority of the study months, in agreement with the hierarchical clustering.

## Discussion

Meat inspection generates a continuous stream of data that can be a source of information for syndromic surveillance, benefiting livestock and poultry surveillance with relevance to health, welfare and commercial productivity. In this study, we have detected relational trends in nine months of meat inspection records collected from broiler chickens over a three-year period that could be applied as surveillance indicators.

Patterns consistent with the presence of infection-related processes were identified in broiler populations at slaughter using correlation-based hierarchical clustering. Identification of specific signs that might be indicative of disease presence is the main objective of syndromic surveillance [12]. For example, a cluster of five conditions: whole carcass condemnations for pericarditis, perihepatitis, peritonitis, liver only and heart only, can be related to infectious processes such as colibacillosis or introduction of an avian hepatitis virus [32, 33]. Here, the clustering of these five conditions identified in May 2016 could be a surveillance indicator signalling an increase of an endemic condition or introduction of a new disease into the broiler population. Similarly, a clustering pattern including whole carcass condemnation for perihepatitis and liver only condemnations detected in most of the studied months could indicate the presence of endemic infectious processes like necrotic enteritis or immunosuppressive pathogens such as infectious bursal disease virus (IBDV) or chicken anaemia virus (CAV) [34, 35]. A further investigation into disease presence or incursion and its risk factors can be informed by detection of such specific clusters of conditions. Previous studies have identified relationships between broiler condemnations and risk factors at different levels of the production chain including chicken, farm, transport and slaughterhouse variables [36–38]. Now, detection of variation in condemnation reasons through their clustering can be relevant for on-farm decision making in subsequent flocks to address the most likely production chain risk factors.

Relationships between some condemnation categories were identified by both correlation-based cluster and association rules analyses, demonstrating the complementary nature of the methods. Detection of patterns in agreement between methods could provide more specific targets for investigation. For example, conditions such as ascites, abnormal colour, hard breast and tumours were correlated by hierarchical cluster analysis and associated in frequent association rules, supporting a common biological explanation. Hard breast, a breast muscle myopathy, and ascites, a metabolic condition associated with the genetic selection of broilers for rapid growth, could both be influenced by shared genetic factors [39, 40]. Ascites, an accumulation of non-inflammatory transudate in the abdominal cavity and other internal spaces, can be a sign of circulatory deficiency or dysfunction of multiple body systems [41]. Such deficiencies can exacerbate the consequences of infection by pathogens associated with condemnation for abnormal colour or tumours. The complex etiology of ascites could explain its strong presence in the majority of association rules. Although it is not possible to draw etiological conclusions from correlations and associations between condemnations identified by the two methods, the information could be used to generate hypotheses and direct further investigation.

Association rules analysis identified two types of patterns with relevance to broiler population health: a change in the total number of strongly associated rules between months (Fig. 7), and an increase in conditional probability between condemnation categories within the rules. The total number of association rules replicated the pattern of incidence of all-cause monthly condemnation, agreeing with information offered by condemnation monitoring (Fig. 4) for all months except May 2016. The similarity between all-cause monthly condemnation incidence and the total number of association rules could be explained by the high prevalence of ascites and abnormal colour condemnation categories. The observed seasonality of these two conditions was previously linked to temperature extremes which are likely when comparing January and September, as used for analysis [39, 42]. Assessing the number of association rules produced each month contributed an additional measure of incidence and was in agreement with trends detected by cluster analyses. The value of monitoring the total number of association rules has not been reported before. Previous research using association rules analysis has been concerned with interpreting individual rules rather than concluding overall trends of association [28, 43, 44]. Thus, the new and unexpected patterns of association detected here could signal previously unsuspected health issues, suggesting potential value as non-specific surveillance indicators.

The second pattern considered was a comparison of the number of stable association rules that increased their strength of association between the studied months. The longitudinal application of association rules analysis to monitor change in individual rules’ strength has been tested previously with hospital data including a range of outcomes and explanatory variables [45, 46]. In our study, only outcomes data were used for analysis, employing readily available meat inspection data which could have limited the number of informative associations. However, detecting the greatest number of rules with an increased strength of association in May 2016 corroborated findings from the cluster analyses. Thus, to our knowledge, this is an informative and novel finding on the value of association rules for analysis of outcome-only data. This non-specific pattern indicated that several condemnation categories increased their likelihood of being simultaneously present in slaughtered broiler flocks, warranting further investigation.

In *k*-means cluster analysis, a change in frequency of condemnation of some broiler batches was detected in some months. For example, in May 2016 a group of batches was identified in which, on further investigation, a high number of chickens were condemned for dermatitis, hepatitis, farm other, and joint lesions, being distinctly different from the rest of the slaughtered broiler population. These conditions were also clustered by the hierarchical clustering method (Fig. 6). The *k*-means clustering, in contrast to association rules and hierarchical clustering which deal with association of condemnation reasons, grouped broiler batches based on their condemnation frequency. Identification of specific broiler batches with a given frequency of condemnation could therefore provide useful information for epidemiological investigation.

### Summary of the approach

We have presented an approach that was based on detection of unspecified *a priori* patterns in routinely available meat inspection data. Similar to other systems such as the Dutch Cattle Health Surveillance System, we carried out analysis at regular time intervals to capitalise on the value of continuity in the detection of animal health-related trends [47]. We detected two types of patterns: syndrome-related and non-specific. Syndrome-related indicators identified using correlation-based hierarchical clustering, similar to those in the study by Dupuy et al., were groups of condemnation categories that could indicate signs of disease-related processes or signify the presence of associated risk factors [19]. Specific examples discussed here can be related to infectious disease processes or host genetic factors. Meanwhile, patterns detected by association rules and *k*-means cluster analyses were closer to trend detection that could be followed by in-depth analysis such as demonstrated by the Dutch system [47].

Relational patterns detected in conditions co-diagnosed at slaughter provided additional information to that available from monitoring of condemnation incidence. The indicators detected in this study may be more sensitive to changes in incidence levels, or when surveillance is case-specific, signalling changes in prevalence of endemic conditions [13, 48]. The high sensitivity of the surveillance system might be especially relevant for use on abattoir data where healthy animals are expected to be present for slaughter. Thus, it could be speculated that detected indicators could signal about changes in endemic low prevalence conditions, which are difficult to identify using condemnation incidence monitoring.

Detection of non-specific surveillance indicators can provide immediate value for broiler production, informing decision making along the production chain. Additionally, if implemented in real-time, the analysis demonstrated here could provide feedback to animal health and production experts, defining the health status of broiler populations [15]. Finally, with the accumulation of longitudinal information, detection of patterns in meat inspection data can help define objectives for prospective surveillance systems and focus monitoring on trends that are relevant to health status [9].

### Limitations

There were a number of limitations and assumptions in the analyses described here. The conclusions reached were limited to data from one broiler integrator. Extrapolation to the national level should be done with caution. However, the use of data generated in a single slaughterhouse addressed the issue of standardisation of broiler abattoir findings between slaughterhouses and possibly increased the specificity of patterns [49].

It was assumed that the broiler abattoir data provided a sufficient level of detail to extract informative data structures. Previous research suggested higher specificity of data provided by reports of partial carcass condemnation, while whole carcass condemnation data were also concluded relevant to animal health, showing better detection of known disease status in some studies [16, 19, 38, 50]. Thus, it was assumed that broiler condemnation data recorded at a batch level and presenting a range of whole carcass condemnation reasons are comparable to partial condemnation data of large farm animals.

The three unsupervised machine learning methods used in this study could have been affected by the degree of subjectivity in the selection of thresholds. However, to minimise this, and in addition to adhering to the established methodologies and considering the biological plausibility of associations, all meat inspection records were used for the analysed months rather than a sample of data to ensure the representativeness of the discovered patterns.

Finally, no data were available to validate the relational patterns detected here against a confirmed outbreak or a known disease status. Thus, future studies are needed to validate patterns identified against confirmed diagnoses, and to establish the practical implications of patterns detected for decision making in broiler production settings. For instance, the study findings could be used to inform the production model for the selection of tools to address broiler health concerns that are associated with current condemnation.

## Conclusions

This study demonstrated detection of patterns in meat inspection data by applying unsupervised machine learning algorithms to routinely collected data using one month intervals. We concluded that the patterns detected were relevant as syndromic surveillance indicators. Detection of patterns in meat inspection data at regular time intervals can be used to offer a more targeted approach to health and welfare management in meat producing animals.

## Data Availability

The data that support the findings of this study are available from the broiler integrator company but restrictions apply to the availability of these data, which were used under license for the current study, and so are not publicly available. Data are however available from the authors (sbuzdugan1@rvc.ac.uk) upon reasonable request and with permission of the broiler integrator company.

## Abbreviations

GB: Great Britain
SyS: Syndromic surveillance
FSA: Food Standards Agency
ABN: Abnormal colour
AST: Ascites
BRU: Bruising
CEL: Cellulitis
DOA: Dead On Arrival
DER: Dermatitis
EMA: Emaciation
EVR: Evisceration Runts
FOL: Foliculitis
HB: Heard Breast
HEP: Hepatitis
IR: Intake runts
JND: Jaundice
JNT: Joint lesions
Prtheart: Partial - Hearts
Prtliv: Partial - Livers
PCS: Pericarditis
PHS: Perihepatitis
PTS: Peritonitis
FOT: Other farm
RES: Respiratory conditions
SKN: Skin conditions
TUM: Tumours
